# Influence of Psychological Nursing Intervention on Psychological State, Treatment Compliance, and Immune Function of Postoperative Patients with Rectal Cancer

**DOI:** 10.1155/2021/1071490

**Published:** 2021-07-19

**Authors:** Xicai Sun, Weifeng Zhong, Jingjing Lu, Wenzhen Zhuang

**Affiliations:** ^1^Department of Health Management, Weifang People's Hospital, Weifang 261041, China; ^2^Hospital Infection-Control Department, Zhangqiu District People's Hospital, Jinan 250200, China; ^3^Department of Geriatrics (II), Qingdao Mental Health Center, Qingdao Seventh People's Hospital, Qingdao 266034, China; ^4^Medical Record Management Section, Weifang People's Hospital, Weifang 261041, China

## Abstract

In order to explore the clinical effect of psychological nursing intervention on postoperative chemotherapy for rectal cancer, 120 cases of rectal cancer patients were selected as the research subjects. The control group received conventional nursing treatment after operation, and the research group received comprehensive psychological nursing intervention on this basis. The self-rating anxiety scale (SAS) scores, self-rating depression scale (SDS) scores, hope level scores, nursing satisfaction, mental state changes, treatment compliance, and immune function of two groups were analyzed and compared. There was no significant difference between the two groups of patients in the preoperative SAS, SDS, and hope level scale scores. After the intervention, postoperative SAS and SDS scores and CD8^+^ value of the research group were significantly lower than those of the control group. In contrast, the postoperative hope level score, treatment compliance, and postoperative CD4^+^/CD8^+^ of the research group were significantly higher, and the nursing satisfaction was better than that of the control group. The application of psychological nursing intervention in postoperative chemotherapy for patients with rectal cancer can effectively relieve anxiety and depression of patients, promote patients to establish a healthy and coordinated mental state, improve treatment compliance, improve immune function, and promote disease recovery.

## 1. Introduction

Rectal cancer is a relatively common type of gastrointestinal malignant tumor, which has clinical treatment difficulties such as high incidence, difficult operation, and many postoperative complications [1]. According to the latest global cancer burden data released by the International Agency for Research on Cancer (IARC) of the World Health Organization in 2020, more than 1.93 million people worldwide were diagnosed with colorectal cancer in 2020, and the cumulative number of deaths reached 930,000 cases, which confirmed that the mortality rate of colorectal cancer ranks third in the mortality rate of malignant tumors [2]. The induction of rectal cancer is caused by many life factors and genetic factors [3]. Factors such as high protein, high fat, low fiber, low vitamins, and excessive intake of sulfite compounds, obesity, and related family genetic diseases are all important factors that promote the induction of rectal cancer [4, 5].

At present, surgical treatment is still the first choice for the treatment of rectal cancer [6]; the stoma (Mile surgery) combined with abdominal perineal resection is often used for patients with low rectal cancer. However, a colon-abdominal stoma changes the patients' normal defecation method, and patients are prone to negative emotions such as depression and anxiety after surgery [7]. Studies have shown that negative emotions such as depression and anxiety affect the immune system, manifested as immunosuppression, and its state is related to tumor growth, metastasis, and other factors [8]. Drastic emotional changes can lead to the disorder of the body's neuroimmune endocrine network, cause the body's immune function disorders, and lose its normal inhibitory effect on the occurrence and development of tumors [9, 10]. Therefore, psychological problems have an adverse effect on the safety, compliance, and immune function of cancer patients [4, 11], and the evaluation of patients' anxiety, depression, and other psychological emotions has received more and more clinical attention [12, 13].

Psychological intervention nursing mainly refers to the use of psychological theory and technology by nursing staff to solve the psychological problems of patients [14, 15]. According to the objects of intervention, it can be divided into psychological treatment, psychological prevention, psychological support, and mental health. Psychotherapy mainly aimed at the diagnosis and early standard treatment of psychological disorders. The goal of treatment is to alleviate the suffering of patients with mental disorders. Psychological prevention is the use of mental health education to guide people with psychological problems. The goal of treatment is to reduce the risk of psychological disorders.

By comparing the treatment status of the patients in the control group and the research group in terms of psychological status, treatment compliance, and immune function, the efficacy of psychological intervention in the comprehensive treatment of rectal cancer patients with neoadjuvant chemotherapy was evaluated in this study. This further lays a solid theoretical foundation for strengthening and standardizing the status and role of supportive psychological intervention treatment in the comprehensive treatment plan for patients with rectal cancer.

## 2. Materials and Methods

### 2.1. Clinical Data

The 120 patients undergoing rectal cancer surgery at Weifang People's Hospital, Weifang, China, from March 2020 to March 2021 were included in this study. The clinical data of 120 patients with rectal cancer undergoing surgical treatment in the hospital were selected and randomly divided into the control group (*n* = 56) and the research group (*n* = 64). The 56 cases in the control group had a male-to-female ratio of 4 : 3, aged from 38 to 78 years, with an average age of 53.29 ± 18.61 years; the education level was 15 cases of junior high school and above, 32 cases of high school and junior college, and 9 cases of bachelor degree and above. The 64 cases in the research group had a male-to-female ratio of 37 : 27, and they were 38–78 years old, with an average age of 52.56 ± 8.25 years; the educational level was 18 cases were junior high school and above, 35 cases were high school and junior college, and 11 cases were bachelor degree or above. There was no statistically significant difference between the two groups of patients in gender, age, and educational level, and they were comparable. This study was approved by the Ethics Committee of the Weifang People's Hospital, Weifang, China.

### 2.2. Inclusion and Exclusion Criteria

Inclusion criteria were as follows. (1) Patients understand the intention of this study and participate voluntarily and signed the “Informed Consent.” (2) The patient is between 38 and 70 years old, and the diagnosis of rectal cancer TNM staging T1∼T2, NO∼N2, and MO. Under rectal cancer Miles surgery, the patient has a basic understanding of the condition. (3) The patient has no heart, kidney, liver, and other dysfunction, no other malignant tumors, no history of thyroid disease, no history of mental illness or family history of mental illness, and no history of special medication. (4) The patient with a junior high school education level or above can correctly understand the content of the questionnaire, answer the question autonomously, and normally listen, speak, read, and write in Chinese language.

Exclusion criteria were as follows. (1) Patients with chemotherapy, radiotherapy, or immunotherapy before Miles surgery; patients with a history of tumor metastasis in the brain; patients with severe dysfunction of the heart, liver, and kidney, hyperthyroidism, or hypothyroidism. (2) Patients with severe medical diseases and those who have a history of severe mental illness or neurosis. (3) Patients who were more than 70 years old or younger than 18 years old and TNM staging >T2 and >N2, patients with adjuvant radiotherapy or chemotherapy, or patients with distant metastases. (4) Those who cannot correctly understand the content of the questionnaire and answer the questions on their own initiative, and the patient has severe communication barriers and cannot normally listen, speak, read, and write in Chinese language. (5) The family members ask for concealment, the patient does not understand the condition, or the patient refuses to receive psychological care.

### 2.3. Nursing Methods

The control group received routine care, including hospital admissions, environmental care, patient introduction to the surgeon, and medication guidance to the patient, and close monitoring of the patient's condition and the occurrence of complications. Once abnormalities occur, they will immediately inform the doctor and deal with it effectively. On this basis, the research group implemented comprehensive, personalized, and comprehensive psychological nursing intervention. The intervention time for both groups of patients was 3 months. The specific measures were as follows.

### 2.4. Psychological Nursing Intervention

Psychological counseling was provided to the treated patients to understand their true inner thoughts and negative emotions in detail and evaluate them. Relevant theoretical knowledge is retrieved in a targeted manner, and questions were answered in a timely manner to eliminate patients' doubts. Psychological intervention methods such as enlightenment, careful conversation, encouragement, and comfort was applied to alleviate further or eliminate the patients' inner worries, instruct patients to master effective methods to adjust bad emotions, prompt patients to adjust their own bad emotions correctly, establish correct cognition, and face the operation with a good attitude.

### 2.5. Cognitive and Behavioral Intervention

According to the patients' awareness of the disease, we used multimedia, text, and pictures to promote the disease and comprehensively and systematically explain to the patient and his family the cause of rectal cancer, the procedure, the abnormal conditions that may occur after the operation, and the corresponding treatment methods. The method of combining procedural muscle relaxation training and physical function training was adopted, 30 min/time, 2 times/week, all under soothing and soft music. We instruct patients to learn psychological coping strategies and behavioral training strategies, such as postoperative exercises, methods of turning over, and precautions related to diet.

### 2.6. Quality of Life Intervention

Patients with rectal cancer are often accompanied by changes in various living habits after surgery, so nursing staff should promptly correct the patients' unhealthy living conditions. For those with sleep disorders, the causes of sleep disorders should be understood and effective solutions should be provided. In view of the changes in bowel habits brought about by colostomy, which seriously affects the patients' physical and psychological state, the patients' diet and self-care methods were instructed to reduce the patients' dependence on nursing staff and family members, improve the patients' quality of life, and help them establish confidence to overcome disease.

### 2.7. Supervisory Feedback

The patients' emotional changes were recorded. Presentation of patient status, effect of preventive interventions, and discussion of improvement measures were performed during daily shift.

## 3. Observation Index

### 3.1. Evaluation Standards

The improvement of the mental status of the two groups was assessed by the SAS and SDS. SAS is based on the results of the Chinese norm. Those with a standard score of less than 50 are considered normal, 50–60 are considered mild anxiety, 61–70 are considered moderate anxiety, and more than 70 are considered severe anxiety. According to the Chinese norm of SDS, the standard cutoff value is 53 points, 53∼62 points for mild depression, 63∼72 points for moderate depression, and 72 points or more for severe depression. During the treatment, the patient strictly abides by the doctor's advice and adheres to standard treatment as complete compliance. During the treatment period, the patient basically follows the doctor's advice for treatment and occasionally does not receive standard treatment as the basic compliance. During the period, the patient does not strictly follow the doctor's advice for treatment, and the treatment is interrupted as noncompliance. Compliance = (complete compliance + basic compliance)/total number of cases × 100%. The two groups of immune function indicators were recorded and compared, mainly including CD4^+^, CD8^+^, and CD4^+^/CD8^+^.

### 3.2. Nursing Satisfaction Evaluation

According to the detailed rules of nursing satisfaction evaluation, the basis of satisfaction scoring was formulated in combination with the stoma of patients with colorectal cancer. The content includes whether the patients' bowel movement is smooth, whether the bedding is changed in time, whether the nurse's attitude is enthusiastic and positive, and whether the nurse often communicates with the patient. A score of >84 indicates very satisfied, a score of 60–84 indicates basic satisfaction, and a score below 60 indicates dissatisfaction. The treatment satisfaction of the two groups of patients was evaluated after the operation. Total nursing satisfaction = (very satisfied + basic satisfaction)/total number of cases × 100%.

### 3.3. Hope Level Evaluation

The Herth Hope Index (HHI) was used to evaluate the patients' hope in life. The scale includes three dimensions: a positive attitude toward reality and the future, an attitude to take positive actions, and an attitude to maintain an intimate relationship with others. It is scored by four levels, namely, strongly disagree, disagree, agree, and strongly agree, and the total score is 12–48; the higher the score, the higher the level of hope. 12–23 are divided into the low level; 24–35 are divided into the medium level; and 36–48 are divided into the high level. The hope levels of the two groups of patients were evaluated before and after the operation.

### 3.4. Statistical Analysis

The data were all analyzed by SPSS 20.0 statistical software, and the normal measurement data were described in the form of mean ± standard deviation $\left(\bar{x}\pm s\right)$. The comparison of the two groups of normal measurement data between groups and within groups adopts the *t* test; the count data are described in the form of frequency and percentage *n*(%), and the comparison of count data *n*(%) adopts the *χ*^2^ test. *P* < 0.05 means that the difference is statistically significant.

## 4. Results

### 4.1. Comparison of General Information of the Two Groups of Patients

There are 64 patients in the research group, and the male-to-female ratio is 37 : 27. There were 56 cases in the control group, and the ratio of male-to-female was 4 : 3. The age of the included patients fluctuated between 35 and 78 years old. There was no significant difference in gender between the two groups (*P* > 0.05). In addition, there was no statistically significant difference between the two groups of patients in terms of general information such as gender, age, education, and marital status (*P* > 0.05). As given in Table 1, it describes that the general baseline data of the two groups of patients are basically the same.

### 4.2. Changes in the Mental State of the Two Groups

**Figure 1:**
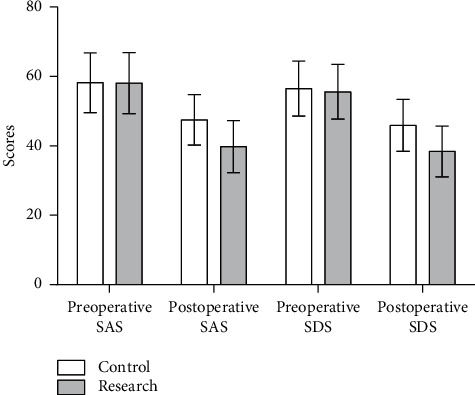
Comparison of SAS and SDS scores between the two groups.

There was no statistically significant difference in the SAS and SDS scores of the two groups of patients upon admission (*P* > 0.05). After the intervention, the SAS and SDS scores of the research group were significantly lower than those of the control group, and the difference was statistically significant (*P* < 0.05), as given in Table 2 and Figure 1.

### 4.3. Comparison of Nursing Satisfaction between the Two Groups

Through psychological nursing intervention, the satisfaction degree of the research group reached 96.88%, which was significantly better than that of the control group (80.36%), and the difference was statistically significant (Table 3) (*P* < 0.05).

### 4.4. Two Groups of Hope Level Evaluation

There was no significant difference in the preoperative HHI score between the two groups (*P* > 0.05). The postoperative research group's attitude towards reality and the future, the attitude of taking positive actions, and the attitude of other people's intimacy and the total score were significantly higher than those of the control group, and the difference was statistically significant (Table 4) (*P* < 0.05).

### 4.5. Two Sets of Compliance

The compliance of the research group and the control group is given in Table 5. The treatment compliance of the research group (89.06%) is higher than that of the control group (71.43%).

### 4.6. Two Groups of Immune Function

The immune function indexes of the research group and the control group are given in Table 6. After the intervention, CD4^+^ and CD4^+^/CD8^+^ of the research group were higher than those of the control group.

## 5. Discussion

Rectal cancer is a serious life-threatening disease, and patients are prone to severe psychological stress. In addition, patients' lifestyles and habits often undergo major changes after surgery, and patients are prone to negative emotions such as depression and anxiety. If this emotion is not properly adjusted, it will have adverse effects on the patients' physiology, immunity, and social activities. It is easy for the patients to lose confidence and be unable to actively cooperate with the doctor's treatment, which affects the compliance of treatment and the quality of life and reduces the survival rate of the patients.

According to the results of the study, the changes in the psychological state of the patients in the research group, the evaluation of satisfaction with care, the evaluation of hope level, and the compliance with treatment were all significantly higher than before, and the immunity of the patients was also enhanced compared with before. In comparison between the groups, the evaluation of treatment compliance, satisfaction with care, and hope level of patients in the research group was significantly higher than that of the control group, and the difference was statistically significant. Through the detection of relevant indicators of the patients' immune function, the results showed that there was no significant difference in the indicators of peripheral blood T cell subsets between the two groups of patients before surgery. After the psychological nursing intervention, the research group's CD4^+^, CD8^+^, and CD4^+^/CD8^+^ all changed significantly compared with before the intervention, and the ratio of CD4^+^ to CD4^+^/CD8^+^ was significantly higher than that of the control group.

To sum up, in the treatment of patients with rectal cancer and colostomy, psychological nursing interventions for different treatment stages of the disease can effectively reduce the patients' anxiety and depression, improve patients' compliance with treatment, and enhance the patients' immune function. It also can promote patients to establish a healthy and coordinated mental state, build confidence in life, improve the level of hope, improve nursing satisfaction, and promote recovery. However, due to the limitations of the environment, sample, time, and other factors, the survival prognosis of the two groups of patients has not been analyzed, and further studies are needed to supplement it.

## Figures and Tables

**Table 1:** Comparison of general information of subjects in the intervention group and the control group, *n*(%).

Variables	Control (*n* = 56)	Research (*n* = 64)	*t*/*χ*^2^	*P*
Gender			0.368	0.926
Male	32 (57.14)	37 (57.81)		
Female	24 (42.86)	27 (42.19)		

Age (years)	53.29 ± 8.61	52.56 ± 8.25	0.429	0.713
<40	4 (7.14)	6 (9.37)		
40∼50	12 (21.42)	18 (28.13)		
51∼60	20 (35.72)	22 (34.37)		
>60	20 (35.72)	18 (28.13)		

Education			0.942	0.246
Junior high school and above	15 (26.79)	18 (28.12)		
High school and junior college	32 (57.14)	35 (54.69)		
Bachelor degree or above	9 (16.07)	11 (17.19)		

Marriage status			1.946	0.425
Unmarried	4 (7.14)	4 (6.25)		
Married	46 (82.14)	50 (78.13)		
Divorced	6 (10.72)	10 (15.62)		

**Table 2:** Comparison of SAS and SDS scores between the two groups.

Grading index	Scoring stage	Control	Research	*t*	*P*
SAS	Preoperation	58.2 ± 8.6	58.1 ± 8.8	0.35	0.625
	Postoperative	47.5 ± 7.3	39.8 ± 7.5	6.24	0.035

SDS	Preoperation	56.5 ± 7.9	55.6 ± 7.9	0.29	0.158
	Postoperative	45.9 ± 7.5	38.4 ± 7.3	7.01	0.022

**Table 3:** Comparison of SAS and SDS scores between the two groups before and after nursing *n* (%).

Group	Case	Very satisfied	Basic satisfaction	Dissatisfaction	Total satisfaction
Research	64	33 (51.56)	29 (45.31)	2 (3.13)	62 (96.88)
Control	56	24 (42.86)	21 (37.50)	11 (19.64)	45 (80.36)
*χ* ^2^					6.124
*P*					0.014

**Table 4:** Comparison of HHI scores between the two groups $\left(\bar{x}\pm s\right)$.

Group	Attitude towards reality and the future		Attitude of taking positive actions		Attitude of other people's intimacy		Total score	
	Preoperation	Postoperative	Preoperation	Postoperative	Preoperation	Postoperative	Preoperation	Postoperative
Research	7.38 ± 1.36	12.11 ± 1.72	8.69 ± 1.78	13.81 ± 1.49	8.04 ± 1.92	13.82 ± 1.86	24.55 ± 2.18	39.85 ± 2.24
Control	7.04 ± 1.31	10.99 ± 1.38	8.82 ± 1.66	11.34 ± 1.56	7.96 ± 1.98	12.56 ± 1.88	24.13 ± 2.11	34.62 ± 2.16
*t*	−0.871	−2.972	0.245	−5.956	0.069	−2.561	−0.278	−7.922
*P*	0.554	0.038	0.721	0.011	1.116	0.024	0.714	0.008

**Table 5:** Compliance comparison between the two groups, *n*(%).

Group	Case	Complete compliance	Basic compliance	Noncompliance	Total compliance
Research	64	31 (48.44)	26 (40.63)	7 (10.93)	57 (89.06)
Control	56	16 (28.57)	24 (42.86)	16 (28.57)	40 (71.43)
*χ* ^2^					5.05
*P*					0.032

**Table 6:** Comparison of immune function between the two groups $\left(\bar{x}\pm s\right)$.

Scoring index	Scoring stage	Control	Research	*t*	*P*
CD4^*+*^	Before intervention	38.3 ± 7.26	38.1 ± 7.32	0.24	0.152
	After intervention	39.5 ± 8.01	46.9 ± 7.95	5.91	0.031

CD8^*+*^	Before intervention	37.2 ± 7.44	36.8 ± 7.06	0.21	0.445
	After intervention	37.8 ± 7.52	31.2 ± 7.13	3.98	0.021

CD4^+^/CD8^+^	Before intervention	1.03 ± 0.28	1.03 ± 0.24	0	0.351
	After intervention	1.09 ± 0.29	1.5 ± 0.34	7.51	0.015

## Data Availability

All data generated or analyzed during this study are included in the published article. The datasets used and/or analyzed during the present study are available from the corresponding author on reasonable request.
